# Correlation between spasticity and corticospinal/corticoreticular tract status in stroke patients after early stage

**DOI:** 10.1097/MD.0000000000033604

**Published:** 2023-04-28

**Authors:** Min Jye Cho, Sang Seok Yeo, Sung Jun Lee, Sung Ho Jang

**Affiliations:** a Department of Physical Medicine and Rehabilitation, College of Medicine, Yeungnam University, Namku, Taegu, Republic of Korea; b Department of Physical Therapy, College of Health Sciences, Dankook University, Dongnamgu, Cheonan, Republic of Korea.

**Keywords:** corticoreticular tract, corticospinal tract, diffusion tensor imaging, modified Ashworth scale, spasticity

## Abstract

We investigated the correlation between spasticity and the states of the corticospinal tract (CST) and corticoreticular tract (CRT) in stroke patients after early stage. Thirty-eight stroke patients and 26 healthy control subjects were recruited. The modified Ashworth scale (MAS) scale after the early stage (more than 1 month after onset) was used to determine the spasticity state of the stroke patients. Fractional anisotropy (FA), apparent diffusion coefficient (ADC), fiber number (FN), and ipsilesional/contra-lesional ratios for diffusion tensor tractography (DTT) parameters of the CST and CRT after the early stage were measured in both ipsi- and contra-lesional hemispheres. This study was conducted retrospectively. The FA and FN CST-ratios in the patient group were significantly lower than those of the control group (*P* < .05), except for the ADC CST-ratio (*P > *.05). Regarding the DTT parameters of the CRT-ratio, the patient group FN value was significantly lower than that of the control group (*P* < .05), whereas the FA and ADC CRT-ratios did not show significant differences between the patient and control groups (*P > *.05). MAS scores showed a strong positive correlation with the ADC CRT-ratio (*P* < .05) and a moderate negative correlation with the FN CRT-ratio (*P* < .05). We observed that the injury severities of the CST and CRT were related to spasticity severity in chronic stroke patients; moreover, compared to the CST, CRT status was more closely related to spasticity severity.

## 1. Introduction

Spasticity is defined as a velocity-dependent increase in muscle tone characterized by a hyperactive stretch reflex following central nervous system injury.^[1,2]^ Spasticity occurs in up to 65% of stroke patients and is closely related to poor motor function, including dexterity loss, muscle weakness, contracture, and pain.^[3–9]^ Several mechanisms involved in the pathophysiology of spasticity have been suggested, including abnormal sensory processing (changes in the balance of excitatory and inhibitory inputs) in the intraspinal network, decreased post-activation depression (a phenomenon that controls excitability of the stretch reflex), muscle immobilization at short lengths (leads to muscle contracture, and contributes to hypertonia), and abnormal supraspinal influences due to injury of neural tracts such as the corticospinal tract (CST), corticoreticulospinal tract, and vestibulospinal tract (VST).^[10–14]^ However, to date, a precise description of the pathophysiologic mechanism of spasticity has not been fully elucidated.

Among various descending motor pathways, the CST, the corticoreticulospinal tract comprising the corticoreticular tract (CRT) and the medial and lateral reticulospinal tracts (RSTs), and the VST have been suggested to be associated with spasticity.^[10–15]^ Among these neural tracts, the CST and the CRT with the lateral RST are reported to provide inhibitory inputs to the intraspinal network, acting as a supraspinal inhibitory system.^[10–15]^ In contrast, the medial RST and VST provide excitatory inputs to the intraspinal network, functioning as a supraspinal excitatory system.^[10–15]^ Previous studies have detected correlations between spasticity and the neural tracts originating from the brainstem, such as the RST and VST, by using neurophysiological methods.^[14,16–19]^ However, little is known about the correlation between spasticity and other neural tracts for motor function, such as the CST and CRT.^[14]^

The introduction of diffusion tensor tractography (DTT), which is derived from diffusion tensor imaging (DTI), has enabled 3-dimensional reconstruction and estimation of various neural tracts, including the CST, CRT, and VST.^[20–23]^ Consequently, several studies have reported on CST, CRT, and VST injuries in stroke patients with impaired motor function.^[24–31]^ However, no DTT-based study on the correlation between spasticity and the above neural tracts in stroke patients has been reported. Among the above-mentioned neural tracts, previous studies have reported that the VST has a minor role in spasticity.^[10–13]^ In this study, we hypothesized that spasticity in stroke patients could be related to CST and CRT injuries.

In the current study, by using DTT, we investigated the correlation between spasticity and the state of the CST and CRT in stroke patients after early stage.

## 2. Materials and methods

### 2.1. Subjects

In this study, the stroke patients were recruited according to the following inclusion criteria: first-ever stroke; age at the onset of stroke: 20 to 69 years; spontaneous intracerebral hemorrhage or cerebral infarction confined to a unilateral hemisphere, as confirmed by a neuroradiologist; DTI scan performed after the early stage (more than 1 month after onset); spasticity in the contra-lesional extremity (modified Ashworth scale [MAS] score after the early stage ≥ 1^[32,33]^); no history of neurologic/psychiatric disease or head trauma. MAS scores, used to determine the spasticity state of the stroke patients, were obtained at the time of DTI scanning.^[32,33]^ This study was conducted retrospectively, and all patients and control subjects provided written informed consent. The study protocol was approved by the institutional review board of a university hospital (IRB number: YUMC 2021-03-014).

### 2.2. DTI and tractography

**Figure 1.**
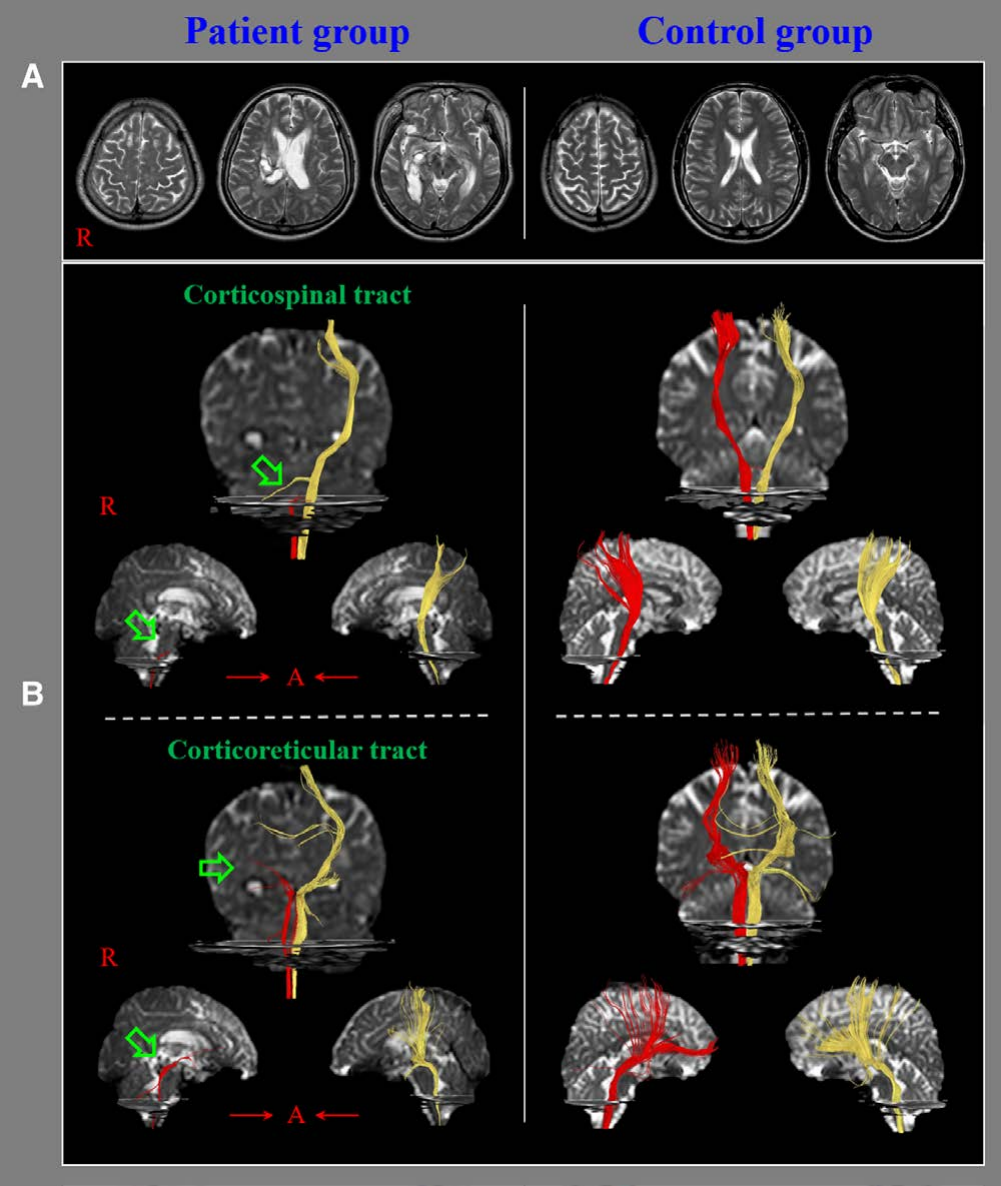
(A) T2-weighted brain magnetic resonance images at the time of diffusion tensor imaging in representative subjects of the patient (56-yr-old male) and control (62-yr-old male) groups. (B) Results of diffusion tensor tractography of the corticospinal tract (CST) and corticoreticular tract (CRT): the ipsilesional CST and CRT of the patient showed discontinuations at the brainstem and subcortical white matter (arrows), respectively, with no such discontinuations in the control subject.

The DTI data were acquired at an average of 10.53 ± 10.90 months after stroke onset using a 1.5 T Philips Gyroscan Intera system (Philips, Ltd, Best, Netherlands) equipped with a Synergy-L Sensitivity Encoding (SENSE) head coil and using a single-shot, spin-echo planar imaging pulse sequence. For each of 32 non-collinear diffusion sensitizing gradients, 60 contiguous slices were acquired parallel to the anterior commissure-posterior commissure line. Imaging parameters were as follows: acquisition matrix = 96 × 96, reconstructed to matrix = 192 × 192, field of view = 240 mm × 240 mm, TR = 10,398 ms, TE = 72 ms, parallel imaging reduction factor (SENSE factor) = 2, EPI factor = 59 and b = 1000 s/mm^2^, NEX = 1, thickness = 2.5 mm. Fiber tracking was performed by applying the fiber assignment continuous tracking algorithm implemented within DTI task card software (Philips Extended MR WorkSpace 2.6.3). Each DTI replication was intra-registered to baseline “b0” images to correct for residual eddy-current image distortions and head motion effects by using a diffusion registration package (Philips Medical Systems). The CST was reconstructed using fibers passing through 2 regions of interest (ROIs) on the DTI color map. The seed ROI was placed at the upper pons, and the target ROI was placed at the mid pons.^[23]^ For analysis of the CRT, the seed ROI was placed on reticular formation of medulla, and the target ROI was placed on the midbrain tegmentum.^[21]^ Termination criteria used for fiber tracking were fractional anisotropy (FA) < 0.15 and angle < 27°.^[34]^ The FA, apparent diffusion coefficient (ADC), and fiber number (FN) values for the CST and CRT after the early stage were measured in both the ipsilesional and contra-lesional hemispheres. Subsequently, ipsilesional/contra-lesional ratios of the CST and CRT for each of the DTT parameters (FA, ADC, and FN) after the early stage were calculated and are presented as CST- and CRT-ratios, respectively (Fig. 1).

### 2.3. Statistical analysis

SPSS software (version 21.0, SPSS Inc., Chicago, IL) was used for data analysis. The chi-squared test was used to assess a sex-based difference between the patient and control groups. Independent *t* tests were used to examine the age distribution difference and the DTT parameter differences in CST- and CRT-ratios between the patient and control groups. The level of statistical significance was set at *P* < .05. The MAS scale category 1 + was modified to a score of 1.5 for statistical analysis purposes. Spearman rank correlation coefficients were used to examine correlations between MAS score and CST- and CRT-ratios for each of the DTT parameters. A correlation coefficient (*R*-value) was interpreted as strong when >0.50, as moderate when between 0.30 and 0.49, and weak when between 0.10 and 0.29.^[35]^

## 3. Results

Thirty-eight stroke patients (23 males, 15 females; mean age 52.61 ± 12.18 years; age range 24–69 years; 19 intracerebral hemorrhage and 19 cerebral infarction) and 26 age- and sex-matched healthy control subjects (15 males, 11 females; mean age 49.00 ± 13.00 years; age range 26–77 years) with no history of neurologic/psychiatric disease or head trauma were recruited. Demographic data for the patient and control groups are summarized in Table 1. No significant differences in age or sex distributions were observed between the patient and control groups (*P* > .05).

**Table 1:** Demographic and clinical data for the patient and control groups.

	Patient group	Control group
Age (yr)	52.61 (12.18)	49.00 (13.00)
Sex, male/female	23/15	15/11
Injury mechanism: ICH/Cerebral infarction	19/19	
MAS	1.87 ± 0.80	

ICH = intracerebral hemorrhage, MAS = modified Ashworth Scale.

The CST- and CRT-ratios for each DTT parameter in the patient and control groups are summarized in Table 2. The FA and FN CST-ratios of the patient group were significantly lower than those of the control group (*P* < .05). However, there was no significant difference between the 2 groups in the ADC CST-ratio (*P* > .05). Regarding the CRT, the FN CRT-ratio of the patient group was significantly lower than that of the control group (*P* < .05), whereas the FA and ADC CRT-ratios were not significantly different between the patient and control groups (*P* > .05).

**Table 2:** Comparison of ipsilesional/contra-lesional ratios of the corticospinal and corticoreticular tracts for diffusion tensor tractography parameters between the patient and control groups.

		CST-ratio		CRT-ratio		
	FA	ADC	FN	FA	ADC	FN
Patient group	0.88 (0.13)	1.04 (0.10)	1.04 (0.26)	0.96 (0.06)	1.04 (0.12)	0.68 (0.32)
Control group	0.97 (0.03)	1.01 (0.04)	0.34 (0.28)	0.97 (0.04)	1.01 (0.05)	1.10 (0.43)
*P* value	.001*	.189	.001*	.776	.177	.001*

Values represent means (± standard deviations) ADC = apparent diffusion coefficient, CRT = corticoreticular tract, CST = corticospinal tract, FA = fractional anisotropy, FN = fiber number.

* *P* < .05.

Correlations between MAS score and the CST- and CRT-ratios for the DTT parameters in the patient group are summarized in Table 3. The MAS score showed a strong positive correlation with ADC CRT-ratio (*R* = 0.574, *P* < .05) and a moderate negative correlation with the FN CRT-ratio (r = −0.356, *P* < .05).^[35]^

**Table 3:** Correlation between modified Ashworth scale scores and ipsilesional/contra-lesional ratios of the corticospinal and corticoreticular tracts for diffusion tensor tractography parameters.

	CST-ratio			CRT-ratio		
MAS	FA	ADC	FN	FA	ADC	FN
	0.113	0.135	−0.297	−0.131	.574*	−.356*

ADC = apparent diffusion coefficient, CRT = corticoreticular tract, CST = corticospinal tract, FA = fractional anisotropy, FN = fiber number, MAS = modified Ashworth Scale.

* Significant differences, *P *< .05.

## 4. Discussion

In the present study, by examining the correlation of MAS score with DTT parameter ratios for the CST and CRT, we investigated the correlation between spasticity and the CST and CRT states in stroke patients after early stage. Our results are summarized as follows. First, our CST-ratio examination showed that the patient group had lower FA and FN values than those of the control group. Second, the CRT-ratio assessment showed that the FN value in the patient group was lower than that in the control group. Third, the MAS score had a strong positive correlation with the ADC CRT-ratio and a moderate negative correlation with the FN CRT-ratio.

Among the various DTT parameters that can be examined, FA, ADC, and FN values are most commonly used when evaluating the state of neural tracts in patients with brain injury.^[20,36,37]^ The FA parameter, which indicates the degree of directionality of water diffusion, is used to assess the degree of tract directionality, whereas the ADC parameter indicates the magnitude of the water diffusion.^[20,36,37]^ Therefore, the FA and ADC parameters may be used to indicate the microstructural integrity status of white matter microstructures, such as axons, myelin, and microtubules.^[20,36,37]^ In contrast, the FN value indicates the number of voxels in a neural tract, suggestive of the total number of fibers in a neural tract. Therefore, decrements in FA and FN values and an increment in the ADC value of a neural tract indicate that neural tract injury status.^[20,36,37]^ In addition, DTT parameters presented as ipsi-/contra-lesional ratios of a neural tract reflect the degree of asymmetry between the ipsi- and contra-lesional tracts. Therefore, relatively large decrements in FA and FN values and increment in ADC value in bilateral ratios for a neural tract indicate greater injury in the ipsilesional neural tract than in the contra-lesional tract. Consequently, greater decrements in FA and/or FN CST- and CRT-ratios in the patient group than in the control group indicate the stroke patients exhibit more severe injuries in the ipsilesional CST and CRT than in the contra-lesional tracts.

The relationship between MAS scores and DTT parameter CST- and CRT-ratios in the patient group showed that the DTT parameter CST- ratios had no correlation with the MAS score, whereas, the ADC CRT-ratio was strongly positively correlated with the MAS score, and the FN CRT-ratio was moderately negatively correlated with the MAS score. Compared with the contra-lesional CRT, injury severity of the ipsilesional CRT was significantly related to the severity of spasticity in stroke patients after early stage. Specifically, compared with the ipsilesional CST, DTT results for the ipsilesional CRT were closely associated with spasticity severity.

Among various pathophysiologic mechanisms of spasticity, abnormal supraspinal factors due to injury of neural tracts are reported to be major causes of spasticity.^[10–13,15]^ In detail, spasticity is maintained by controlled balancing of the stretch reflex via inhibitory influences of the CST and the CRT with the lateral RST and the excitatory influences of the medial RST and VST; however, the VST is reported to have a minor effect on spasticity.^[10–13,15]^ Therefore, in brain injuries that include the CST and CRT, the inhibitory influences on the medullary brainstem and intraspinal network can be lost, leading to an unopposed excitatory influence by the medial RST; thus, hyperexcitability of the medial RST can occur.^[10–13,15]^ Hyperexcitability of the RST has been related to spasticity, abnormal motor synergy, and disordered motor control.^[11,18]^ In addition, previous studies have reported that if a CST injury due to extensive cortical damage occurs, injury recovery can use an alternative motor pathway, such as the RST, for compensation, leading to abnormal motor synergy, dexterity loss, and spasticity.^[18,38–41]^ Therefore, although various neural tracts are involved in spasticity, given the above results and the difficulty in identifying the individual actions of brainstem nuclei when using DTT, we focused on CST and CRT injuries in stroke patients after early stage with spasticity.^[12]^ Consequently, based on previous and current studies, we suggest that abnormal supraspinal influences on the intraspinal network due to the CRT injury rather than the CST injury could be responsible for spasticity in stroke patients after early stage.

A few studies have reported on the correlation between spasticity and CST and/or CRT injuries in stroke patients.^[42–44]^ In 2016, Barlow demonstrated injuries of gray (insula, basal ganglia, and thalamus) and white (pontine crossing tract, CST, internal capsule, corona radiate, external capsule, and superior fronto-occipital fasciculus) matters using lesion density plots and voxel-based lesion-symptom mapping in 20 acute patients with spasticity following ischemic stroke.^[42]^ During the same year, Lee et al^[43]^, using brain magnetic resonance imaging and positron emission tomography, reported on a patient who showed spasticity in the left leg due to injuries of the CST and CRT following infarction in the right cerebral peduncle. In 2019, Plantin et al demonstrated CST injury due to intracerebral hemorrhage or cerebral infarction by examining weighted CST lesion loads and voxel-based lesion-symptom mapping in 61 stroke patients with hand spasticity evaluated by the NeuroFlexor method, which accounts for velocity dependence in the tonic stretch reflex.^[44]^ Taken together, our results support previous studies showing that injuries of the CST and/or CRT are associated with spasticity. To the best of our knowledge, this is the first DTI-based study to report a correlation between spasticity and CST and CRT injuries.

Several limitations of this study should be considered. First, the fiber tracking technique applied during DTT reconstruction is operator-dependent. Second, this study recruited a relatively small number of subjects. Third, among the descending motor pathways related to spasticity, we only reconstructed the CST and CRT because the descending motor pathways originating from the brainstem are challenging to reconstruct using DTI. Fourth, only MAS scale was used to evaluate spasticity in this study.

By contrast, the Modified MAS (MMAS) scale which is an advanced version of MAS that was reported as a more reliable and valid method than the MAS scale by excluding the MAS score category 1 + aspect, could be better for this kind of study.^[45–47]^ However, the reliability and validity of Ashworth scale methods for measuring the severity of muscle spasticity have been controversial.^[45,48]^ Therefore, further prospective studies that include larger numbers of patients, more advanced spasticity evaluation tools, and enhanced DTI techniques to reconstruct motor pathways originating from brainstem should be encouraged.

In conclusion, we have demonstrated that CST and CRT injury severities were related to spasticity severity in stroke patients after early stage. In particular, of the 2 tracts assessed, CRT injury status was the most closely related to spasticity severity. Our results suggest that recovery of CST and CRT injuries through neuro-rehabilitation during the stroke recovery phase is important in the prevention of spasticity after the early stage. Thus, DTT-based reconstruction and assessment of the CST and CRT could be helpful when predicting the occurrence of spasticity and planning neuro-rehabilitation treatments that could improve recovery prognosis.

This work was supported by the National Research Foundation of Korea (NRF) grant funded by the Korean Government (MSIP) (No. 2021R1A2B5B01001386).

MJC and SSY contributed equally to this work.

## Author contributions

**Conceptualization:** Min Jye Cho, Sung Jun Lee.

**Data curation:** Min Jye Cho, Sang Seok Yeo, Sung Jun Lee, Sung Ho Jang.

**Formal analysis:** Sung Jun Lee.

**Investigation:** Min Jye Cho, Sang Seok Yeo, Sung Jun Lee.

**Methodology:** Sang Seok Yeo, Sung Ho Jang.

**Supervision:** Sang Seok Yeo, Sung Ho Jang.

**Visualization:** Sung Jun Lee.

**Writing – original draft:** Min Jye Cho, Sung Jun Lee.

**Writing – review & editing:** Sang Seok Yeo, Sung Ho Jang.
